# Spatial separation enhances speech intelligibility but increases listening effort with session-dependent variability in pupillometric measures

**DOI:** 10.3389/fnins.2025.1655826

**Published:** 2025-11-10

**Authors:** Tanvi Thakkar, Jarett Knoepker, Stephen R. Dennison, Joseph P. Roche, Ruth Y. Litovsky

**Affiliations:** 1 Waisman Center, University of Wisconsin-Madison, Madison, WI, United States; 2 Psychology Department, University of Wisconsin-La Crosse, La Crosse, WI, United States; 3 Department of Communication Sciences and Disorders, University of Wisconsin-Madison, Madison, WI, United States; 4 North American Research Lab, MED-EL US, Durham, NC, United States; 5 Department of Surgery, University of Wisconsin-Madison, Madison, WI, United States

**Keywords:** spatial release from masking (SRM), test retest reliability, pupillometry, listening effort, binaural hearing

## Abstract

**Introduction:**

The current understanding of the cognitive load of listening effort has been advanced by combining speech intelligibility and pupillometry measures. However, the reliability of pupil dilation metrics in complex listening scenarios like spatial release from masking (SRM) remains uncertain. This study investigated how spatial separation of sound sources impacts listening effort (via peak pupil dilation, PPD) and speech intelligibility.

**Methods:**

Speech intelligibility and listening effort were simultaneously measured under co-located and symmetric, spatially-separated conditions at varying signal-to-noise ratios (SNRs).

**Results:**

Results showed that although spatial separation improved speech intelligibility, it did not yield a corresponding reduction in listening effort. Instead, listening effort increased as SNR became more challenging. Furthermore, test–retest reliability was moderate-to-high for speech intelligibility but only moderate-to-low for PPD, with greater consistency observed at more challenging SNRs. These results suggest that obtaining stable PPD measures within an SRM paradigm may be difficult to achieve.

**Discussion:**

These findings indicate that obtaining stable PPD measures within an SRM paradigm can be challenging. Test session reliability is weak when combining SRM paradigms with measures of listening effort, which may reduce statistical power due to factors such as sample size, number of trials, and sessions tested. This is further limited by the relatively small and homogeneous sample of young, typical hearing adults. Future studies should include a larger and more diverse participant group to assess the generalizability of these results.

**Clinical trial registration:**

## Introduction

The allocation of mental resources during a listening task is a process influenced by various environmental and individual factors. Numerous variables, including the acoustic environment (e.g., quiet versus noisy), clarity of the auditory signal, and audibility, play critical roles in determining the amount of cognitive effort a listener expends (Wendt et al., 2016; Winn et al., 2015, 2018). Listening effort can be defined as “a deliberate allocation of mental resources to overcome obstacles in goal pursuit when carrying out a task…specifically when tasks involve listening,” as such, task difficulty is a critical factor which shapes listener engagement and cognitive load (Pichora-Fuller et al., 2016). Studies on listening effort reveal that listeners with hearing impairment often experience difficulties that are associated with increased fatigue (Hornsby, 2013; McGarrigle et al., 2014; Nachtegaal et al., 2009; Wang et al., 2018). Compared to individuals with typical hearing (TH), studies have found that individuals with hearing loss who report higher levels of effort and fatigue are more likely to require recovery after work and are more inclined to take sick-leave due to stress-related factors (Alhanbali et al., 2017; Kramer, 2008; Kramer et al., 2006). The subjective perception of elevated effort in complex listening situations has been linked to feelings of social isolation and anxiety among individuals with hearing loss (Hughes et al., 2018). Furthermore, research has shown that the background noise level or degree of sound degradation can play a considerable role in how motivated individuals are when attempting to understand a talker (Winn, 2016). As a result, listening effort has become a valuable area of research for evaluating hearing performance via behavioral measurement through speech intelligibility scores and physiological, or “objective,” measurement through assessment of task-evoked pupillometric changes. These outcome measures require nuanced interpretation, as listeners’ engagement can lead to substantial variations in effort during listening tasks. These variations become pertinent in complex listening situations, where to understand a conversation, individuals may exert different levels of effort based on task difficulty (Zekveld et al., 2010). While the expectation is that speech intelligibility scores should decrease and listening effort should increase with rising task difficulty, this outcome can vary significantly depending on various listener etiologies and characteristics.

### Pupillometry as a metric for listening effort

A common technique for evaluating listening effort is pupillometry, or the measurement of changes in pupil size, which has been shown to be mechanistically related to effortful listening (Kahneman and Beatty, 1967). Studies have shown that the pupil response is regulated by the autonomic nervous system (Bremner, 2009; May et al., 2019; Wang et al., 2016) which plays an important role in maintaining stability and balance in the body. It reflects the interplay between task difficulty, cognitive demand, and motivation (Beatty, 1982; Kahneman and Beatty, 1966). However, due to the complex nature of pupil dilation, strict experimental paradigms are necessary to accurately observe these effects. When tasks reach a level of difficulty where additional effort seems ineffective, participant motivation declines, leading to a decrease in pupil dilation (Ohlenforst et al., 2017; Pichora-Fuller and Schneider, 1998; Wendt et al., 2016). This phenomenon has been observed when pupil dilation is measured concurrently with a speech intelligibility task; pupil dilation tends to increase with decreasing performance when the processing of the task-relevant stimuli exceeds a moderate level of difficulty, after which pupil dilation decreases due to a decline in motivation and engagement (Aston-Jones and Cohen, 2005). A clear distinction should be made between changes due to motivation versus task difficulty: motivation refers to an individual’s willingness or drive to invest effort in a task, whereas task difficulty refers to a response in the experimental manipulation of the task demands (Pichora-Fuller et al., 2016; Peelle, 2018). While motivation and task difficulty can interact (more difficult tasks may lead some listeners to disengage), they are not the same, most listening effort studies primarily manipulate and measure the effects of task difficulty. For example, Aston-Jones and Cohen’s (2005) adaptive gain theory suggests these processes are mediated by the locus coeruleus, which supports the notion that cognitive resources can be allocated in response to both changing task demands and motivational state. Thus, modulation of effort, as shown by prior research, is assumed to arise in response to task demands from experimental manipulation, rather than from direct changes in motivation. For instance, changes in pupil dilation have also been found to be positively correlated with self-reported task difficulty on speech intelligibility performance scores, where increases in perceived task demands are linked to a decrease in speech scores (Zhou et al., 2022a). It is important to note that without direct assessment or manipulation of motivation, it is difficult to fully evaluate the predictions of motivational accounts of listening effort.

Pupillometry is valuable for assessing cognitive and perceptual processes, but reliable measurement requires data from multiple trials. Extended experiments across multiple sessions could introduce variability in participants’ mental states and environmental conditions, despite rigorous control efforts. A delay between test sessions can introduce increasing confounds, so caution is advised when integrating data from different visits into the same analysis. Winn et al. (2018) outlined detailed pupillometry standards, including controlled environments and analytic methods linking pupil dynamics to cognitive load via autonomic processes (effort, attention, arousal). They identified age, health, pediatric assessment challenges, and fatigue/motivation effects as potential key confounders, suggesting that multisession studies are faced with reliability issues from experimental noise and individual variability.

### Spatial release from masking and listening effort

The existing literature has yet to clarify how spatial separation of sound sources influences reliability of speech intelligibility when assessed alongside measures of listening effort. Therefore, it is also important to consider how binaural hearing mechanisms may interact with listening effort in these contexts. Decades of research has shown that listeners benefit from the spatial separation of target speech from background maskers, a phenomenon known as spatial release from masking (SRM). SRM serves as an assessment for hearing in complex listening situations, whereby an enhancement in speech understanding emerges when a target sound and the masker (such as noise or competing speakers) are spatially separated, as opposed to being co-located or emanating from the same direction (Arbogast et al., 2002; Hawley et al., 2004; Kidd et al., 2010). The improvement in speech understanding is achieved through a combination of binaural unmasking, the head shadow effect, and/or monaural spectral cues (Bronkhorst, 2015; Freyman et al., 2002). Further, SRM can be impacted by various manipulated variables such as signal-to-noise ratio (SNR), similarity between target and maskers, hearing status, and attention (Arbogast et al., 2005; Brungart et al., 2001). In most SRM experiments, the target sound originates from the front, while maskers are presented from various locations, either co-located with or separated from the target (Freyman et al., 2002; Hawley et al., 1999). In the spatially separated condition, when the maskers are positioned symmetrically around the head while the target is presented at 0 degrees azimuth, there is minimal access to monaural head shadow cues, leading listeners to rely more on binaural unmasking (Bronkhorst, 2015; Culling et al., 2004; Jones and Litovsky, 2011). Together, these findings explain how spatial separation is critical for reducing the auditory masking of certain speech sounds, and studies have now begun to explore how spatial hearing abilities interact with cognitive variables such as listening effort.

Current research on the relationship between SRM and listening effort, particularly as it relates to systematic measurement at varying SNRs, is limited. Recent studies investigating the impact of spatial separation on listening effort over headphones, have shown that TH individuals often display a “plateau” in pupil dilation growth when an interfering sound, or masker, is presented dichotically, particularly when the masker has good spectral resolution (DeRoy Milvae et al., 2021). For instance, altering the spectral resolution of the interfering talker can result in greater effects on listening effort than changing the spectral resolution of the target. Although a caveat of DeRoy Milvae et al. (2021) is that they used digit stimuli presented via headphones rather than in a free-field environment, their findings highlight the significant impact of target clarity relative to the masker on listening effort in spatial hearing tasks. Specifically, their results suggest that listening effort increases when participants attempt to ignore a clearer interferer. While this study does not directly examine SRM, they highlight related influences of target and masker clarity on listening effort, emphasizing the need for further research that evaluates these factors within binaural hearing contexts.

Assessing speech intelligibility and pupillometric responses in complex auditory environments with parameters like spatial configuration of maskers, SNR, and masker type has significant implications for users of bilateral hearing assistive devices such as those with hearing aids and cochlear implants (CIs). These measures can provide insights into adaptive benefits of listening effort under ecologically valid conditions. A recent study used functional near-infrared spectroscopy (fNIRS) to investigate cortical hemodynamic responses in a binaural unmasking paradigm with CI-simulated speech (Zhou et al., 2022b). The research examined the effects of SNR and masker spatial configuration (diotic vs. dichotic listening) under these conditions. While their study found no significant main effect of masker configuration on cortical activity, it revealed a significant SNR and masker configuration interaction in the left lateral prefrontal cortex. This interaction suggests that cortical processing in this region is sensitive to changes in SNR in a binaural unmasking paradigm. However, it should be noted that an earlier study by Zhou et al. (2022a) found that fNIRS measurements reflect speech processing rather than listening effort, as indicated by an inverse relationship between hemodynamic responses and self-reported task difficulty, and a positive association with speech intelligibility accuracy. Conversely, pupillometry demonstrated the reverse pattern, highlighting its robustness in assessing listening effort. Together, these studies have demonstrated that effortful listening can be observed in binaural experimental paradigms across heterogeneous listener populations (bilateral CI, single-sided deafness with CI, and TH listeners), however it is still unknown whether these outcomes are reliable and valid as it relates to SNR-dependent measures.

The most recent analysis of SRM and listening effort comes from Suveg et al. (2025) who studied adults with unilateral deafness receiving a CI in their deaf ear one year after initial assessment. This design allowed for direct comparison of participants’ performance and listening effort before and after implantation. Importantly, all participants retained normal acoustic hearing in their non-implanted ear, classifying them as individuals with single-sided deafness and a cochlear implant (SSD-CI). The study concluded that speech intelligibility was significantly higher in the symmetric, spatially-separated conditions compared with the co-located testing conditions while pupil dilation demonstrated no corresponding reduction. The authors attributed this finding to a small sample size with large inter-participant variance. One limitation of this study was that SNR was not systematically manipulated and was only investigated in eight SSD-CI patients, however, their findings highlight the role that participant variability plays on a paradigm with combined measures of SRM and listening effort.

One study which explored the influence of multiple test sessions within-subject was Neagu et al. (2023), allowing a look into reliability via intraclass correlations. This study measured listening effort in a speech-in-noise task, with no variations in spatial configurations, but found that mean and peak pupil dilation (PPD) measures were the most reliable features, while growth curve analysis features showed more variability. Further, they highlighted that baseline correction combined with range normalization provided the highest test–retest reliability. Interestingly, SNR did not consistently impact reliability, and pupil responses correlated more strongly with task performance than with self-reported effort.

Thus, questions remain about whether speech intelligibility scores reliably track listening effort via pupil dilation across spatial configurations and how consistently pupillometry reflects effort under varying noise levels. This study assesses test–retest reliability in TH individuals by comparing speech intelligibility and PPD, the latter serving as a representation for cognitive effort during listening tasks, across two sessions. Here, we examine how spatial separation influences these measures across two distinct time points.

The novelty of our approach lies in applying multiple SNR levels to evaluate pupillometric reliability, building on prior work which only tested a single SNR (Burg et al., 2021; Zekveld et al., 2010). Our design clarifies how pupillometry can quantify listening effort across a range of SNR conditions. The primary objectives of this study were: (1) to present, for the first time, findings from an SRM task using outcome measures of speech intelligibility and pupil dilation across multiple noise conditions, and (2) to examine the effects of spatial separation of target speech from maskers on listening effort by assessing the reliability of PPD and SRM across two temporally distinct test sessions (“Visit 1” and “Visit 2”).

Unlike prior spatial hearing studies that controlled for SNR, here we systematically varied SNRs from −12 dB to +9 dB. First, we hypothesized that participants would exhibit higher speech intelligibility and reduced listening effort (measured via PPD) in the symmetric, spatially-separated conditions compared to the co-located condition. We also hypothesized that listening effort measures would reveal maximal benefits from spatial separation (i.e., reduced PPD) at intermediate SNRs, where spatial cues are perceptually available but cognitive demand remains high, eliciting measurable changes in listening effort due to differences in task difficulty.

Second, we hypothesized that speech intelligibility would be consistent across both test sessions. However, we also hypothesized that listening effort measures would show significant test–retest differences, with reduced PPD in Visit 2 versus Visit 1 across spatial conditions, and greater session effects in co-located than symmetric conditions due to competing mechanisms of task difficulty and familiarity.

It should be noted that this study was conducted in young, TH adults, as such, our design is exploratory and methodological in focus. The objective of the current study was to demonstrate how spatial separation modulates listening effort under controlled laboratory conditions. We hope these results will serve as groundwork for future studies, particularly those using clinical populations and materials.

## Methods

Twenty participants were recruited via flyers posted in public locations and were offered compensation for their participation. The final sample included 20 adult participants (8 males, 12 females) with a mean age of 20.3 years (*SD* = 3.02). All participants passed an air conduction pure-tone hearing screening at 20 dB across frequencies of 0.25, 0.50, 1, 2, 4, and 8 kHz. The study received approval from the University of Wisconsin-Madison Institutional Review Board (IRB), and all participants were screened to confirm typical hearing.

### Experimental setup

Testing was conducted in a standard sound booth (IAC Acoustics, IL, USA). Participants were seated at a table with their chin and forehead supported by a headrest to stabilize their head during testing. The table and chair were adjusted to accommodate each participant’s height and position. A computer monitor was mounted on the table approximately 65 cm from the headrest. An eye tracker camera was secured to the table with a desktop mount, positioned 8 cm in front of the monitor. The test room’s illumination was controlled at 93 lux for all participants. To minimize potential confounds arising from variable ambient light, we maintained isoluminant conditions for all participants, this allowed for consistent comparison of pupil size measurements across all noise and spatial configurations. During the post-hoc analysis we implemented a “range normalization” procedure which reflects changes in cognitive demands rather than changes in ambient light. Audio stimuli were emitted from a loudspeaker (Tannoy, Coatbridge, Scotland) placed directly ahead and above the computer monitor (0° azimuth). Pupil size was recorded in pixels using the “Area” setting on an eye tracker (Eyelink 1,000 Plus; SR Research, Ontario, Canada) with a sampling rate of 1,000 Hz.

### Stimuli

Target stimuli consisted of Harvard IEEE sentences spoken by a male talker, and masking stimuli composed of a series of concatenated AZ-Bio two-talker sentences also spoken by a male talker; all stimuli were played at an overall level of 65 decibels (dB) SPL-A. IEEE sentences were prerecorded in our lab using Audacity (Muse Group, Limassol, Cyprus) and the RME Babyface TotalMix (RME, Haimhausen, Germany) for audio recording, which involved configuring microphones and headphones, allowing for consistent sound card settings via pre-loaded configurations, conducting pre-recording checks to optimize the signal-to-noise ratio, and finally capturing and storing the audio data. A total of 272 unique IEEE sentences, spoken by a single male talker, were used as the target stimulus, while 660 concatenated AzBio sentences spoken by two different male talkers from our lab served as maskers, ensuring that the target and masker were always represented by different talkers. In each masker condition, a random segment from the concatenated masker stimuli was played. Stimuli were presented at eight different SNRs: +12, +9, +6, +3, 0, −3, −6, and −9 dB. To obtain positive SNRs, the loudness of the masker was reduced relative to the target stimulus. Conversely, for negative SNRs, the loudness of the target stimulus was lowered compared to the maskers. This adjustment confirmed that the listening levels remained safe at an overall level of 65 dB SPL-A.

The testing included three main listening configurations for the target and maskers: co-located, symmetric left/right, and quiet (no masker). In the co-located configuration, both the target and masker were emitted from the same loudspeaker positioned at 0° azimuth. In the symmetric left/right configuration, the target was played from a loudspeaker at 0° azimuth, and the masking sounds were simultaneously played from two loudspeakers, located at −90° and 90° azimuths. In the quiet configuration, the target was presented alone at 0° azimuth without any masking noise, however, main analyses focused on masked (co-located and symmetric) conditions. This condition served as a baseline but was not included in primary analyses due to fewer number of trials collected for this condition. The stimuli for the experiment were delivered using custom software developed in MATLAB using PsychToolbox version 3 (Mathworks, Natick, MA USA).

### Procedure

Participants completed two test sessions spaced two weeks apart, each lasting approximately 6 hours (h), labeled here as “Visit 1” and “Visit 2.” Each session was divided into two segments with a 20–30 minute break in between. To minimize fatigue, participants were encouraged to take additional breaks as needed. The structure of having two separate sessions was crucial for evaluating the reliability of measures over time. Each session began with six practice trials in both masked conditions (co-located and symmetric) at an SNR of +3 dB. These trials were designed to familiarize participants with identifying target speech amid background noise. During the main test, each listening condition (quiet, co-located, symmetric left/right) was presented at each SNR, totaling 272 sentences per session. Trials were presented in two blocks per condition, each containing eight sentences. The sequence of conditions was randomized for each participant, and each 8-sentence block contained randomly chosen sentences from the entire IEEE-sentence corpus, selected without replacement. For each SNR, trials in co-located and symmetric configurations were presented consecutively, employing a pseudo-random method. The overall design allowed systematic presentation of all conditions within each session while also allowing participants to familiarize themselves with the sound environments they would encounter while minimizing fatigue. In each block, a pseudorandom order of SNR and listening configuration (co-located or symmetric) was presented. Each condition consisted of 8 sentences per block. Cognitive testing and a 30-min break occurred midway through the session. Quiet trials were also interleaved as indicated in Table 1.

**Table 1:** Order of sentence lists and trial conditions.

Test block	Listening configuration/SNR	Sentence #
Practice	Symmetric/3 dB	6
Practice	Co-located/3 dB	6
1	Co-located/3 dB	8
	Symmetric/3 dB	8
2	Symmetric/−9 dB	8
	Co-located/−9 dB	8
3	Co-located/6 dB	8
	Symmetric/6 dB	8
4	Co-located/9 dB	8
	Symmetric/9 dB	8
5	Symmetric/−12 dB	8
	Co-located/−12 dB	8
6	Symmetric/0 dB	8
	Co-located/0 dB	8
7	Quiet	8
8	Symmetric/−6 dB	8
	Co-located/−6 dB	8
9	Co-located/−3 dB	8
	Symmetric/−3 dB	8
Cognitive testing/break time (30 min)		
10	Symmetric/9 dB	8
	Co-located/9 dB	8
11	Quiet	8
12	Co-located/−12 dB	8
	Symmetric/−12 dB	8
13	Co-located/0 dB	8
	Symmetric/0 dB	8
14	Co-located/−6 dB	8
	Symmetric/−6 dB	8
15	Co-located/−9 dB	8
	Symmetric/−9 dB	8
16	Symmetric/6 dB	8
	Co-located/6 dB	8
17	Symmetric/−3 dB	8
	Co-located/−3 dB	8
18	Symmetric/3 dB	8
	Co-located/3 dB	8

Each block indicates the signal-to-noise ratio (SNR), speaker configuration (co-located or symmetric), and number of sentences per condition. Practice and quiet trials are interleaved. A 30-min cognitive testing break occurred after block 9.

During the experimental trials, pupil data from the listeners was continuously recorded, and intelligibility scores were calculated based on the total number of correctly recalled words, from five keywords present in each sentence. Each trial began with a fixation cross displayed on the monitor. The cross changed from gray to white, signaling trial initiation, followed by a silent 2-second(s) period for baseline pupil measurement. After the speech stimulus was played, participants had a 2-s pause before the cross turned green and two beeps signaled them to repeat the sentence aloud. During this 2-s response window, the experimenter recorded total number of correctly recalled keywords. To allow pupil size to return to baseline, a rest period of 10–15 s was included between trials.

This structured test schedule allowed for balanced exposure across conditions while minimizing fatigue. The order of conditions was pseudo-randomized, with symmetric left/right and co-located conditions presented consecutively within each SNR. Breaks were incorporated after approximately half the trials; however, all participants were encouraged to take breaks between test blocks.

### Data pre-processing

Analysis of pupillometry data requires considering several artifacts. Pupil tracks were preprocessed using blink interpolation (80 ms before, 160 ms after) and low-pass filtering with MATLAB’s “smooth” function (Zekveld et al., 2010). To control for individual and trial-specific variability, pupil dilation data were baseline corrected using a subtractive method, whereby each measured sample, whether obtained from the eye-tracking device or after normalization (Binda and Murray, 2015; Steinhauer et al., 2004). Baseline was calculated as the first 1,000 ms of each trial. We employed a two-step normalization procedure: (1) baseline correction with subtraction of each trial’s pre-stimulus 1,000 ms baseline from all subsequent values in the trial, and (2) range normalization by scaling the baseline-corrected pupil data (generated in step 1) within each participant to their response range across all trials, per Neagu et al. (2023). A per-trial subtractive baseline correction and subsequent range normalization was applied over divisive methods to accommodate participant variability (Neagu et al., 2023; Winn et al., 2018).

All pupil tracks were aligned to stimulus offset to determine a maximum “peak” pupil dilation (PPD). PPD was calculated within a 2,500 ms window (500 ms pre-offset, 2,000 ms post-offset), a critical processing window that has consistently been shown to elicit the largest pupil size during the trial for sentence recognition tasks (Zekveld et al., 2010; Winn et al., 2018). Peak pupil dilation and percentage of correctly recalled words were calculated and extracted for each pupil track and subsequently averaged for each participant, within each listening configuration and SNR. To guarantee data integrity, blinks were identified when samples fell below three standard deviations from the mean, and trials with irregular baselines, extreme distortions, or >45% missing data due to blinks or gaze shifts were excluded. The decision to set a high threshold for blink percentage was informed by the correlation between increased blink rates and heightened perceived effort by participants, resulting in potential omission of high-effort trials (Burg et al., 2022).

Discard rates were low across conditions with 0, 0.43, 0.35% discarded for quiet, co-located, and symmetric, respectively, for Visit 1. For Visit 2, discard rates were 0% (quiet), 0.63% (co-located), and 0% (symmetric). Seventy-one trials (0.015%) were excluded from the SRM calculations to compensate for missing entries. For instance, if a participant completed only seven trials in the co-located condition, one trial in the symmetric condition had to be removed to allow for matching data sets.

### Spatial release from masking analysis

A normality test was not necessary for this dataset as we had 20 participants and a total of 5,112 raw data points across all configurations, SNRs, visits, and participants.

Two outcome measures were analyzed: speech intelligibility, measured as rational arcsine units (RAU) of percent correct and PPD (maximum pupil change from baseline). Speech intelligibility scores were transformed into RAU to address the ceiling effects observed for higher SNRs (Studebaker, 1985; Studebaker et al., 1999). The transformation was performed in R using the “asin” and “sqrt” function. To evaluate our first hypothesis, whereby participants will exhibit higher speech intelligibility and reduced PPD in the symmetric condition compared to the co-located condition, as a function of SNR, we modeled the RAU speech intelligibility data and the PPD using two different linear mixed-effects models implemented using the *lme4* package in R (version 4.2.0). Fixed effects included SNR (coded as a continuous variable), Listening Configuration (coded as a categorical variable), and Test Session (Visit 1 vs. Visit 2, coded as a categorical variable), while Participant ID was included as a random effect. Importantly, following best practices in the analysis of pupillometry and behavioral data, the model was applied to data that were averaged across trials for each session, Listening Configuration, SNR, and participant, rather than to individual trial-level data. Averaging across trials preserves the independence of observations and reduces the risk of inflated Type I error or overparameterization, especially in studies with moderate sample sizes and repeated measures designs, in line with best practices (Winn et al., 2018).

Additionally, test–retest reliability was assessed using intraclass correlation coefficients (ICC; range: 0–1) to quantify measurement consistency across sessions. ICCs represent absolute agreement which incorporates both consistency and between-subject variance. A normality test was not necessary for this dataset as we had 20 participants and a total of 5,112 raw data points across all configurations, SNRs, visits, and participants.

### Speech intelligibility model

To evaluate our first hypothesis, whereby participants will exhibit higher speech intelligibility and reduced PPD in the symmetric compared to the co-located configuration, the following linear mixed-effects model was used to analyze speech intelligibility (measured in rationalized arcsine units, RAU):


RAU∼SNR×Listening Configuration×Visit+(1∣ID)

### Listening effort model

Listening effort, indexed by peak pupil dilation, was analyzed using the following linear mixed-effects model:


$$\begin{array}{l} \text{Peak pupil dilation}\;(\mathrm{PPD})∼\mathrm{SNR}\\ \times \text{Listening configuration}\times \text{Visit}+(1|\mathrm{ID}) \end{array}$$

Both models evaluated main effects and all possible interactions among SNR, Listening Configuration, and Visit. Subject-specific random intercepts were included particularly to be able to evaluate SNR-Listening Configuration interactions. All analyses were conducted in R (version 4.2.0) using packages *lme4 and lmerTest*, with degrees of freedom and *p*-values estimated using Satterthwaite’s approximation.

### Test–retest reliability analysis

To evaluate the second hypothesis, we calculated intra-class correlation coefficients (ICCs) between Visit 1 and Visit 2 for both RAU scores and PPD using a two-way consistency model for single measurements. For PPD analysis, we computed ICCs separately for each configuration using the *icc* function in R with parameters model = “twoway,” type = “consistency,” and unit = “single.” This type of model allows for rank-order stability between visits while accounting for systematic mean differences. The same method was applied to speech intelligibility data, yielding distinct ICC estimates for spatial configurations, allowing direct comparison of test–retest reliability between experimental conditions across each SNR. Analyses were performed using R (version 4.2.0) with the *dplyr* and *irr* packages. ICC values were interpreted using established benchmarks: <0.50: Poor reliability, 0.50–0.75: Moderate reliability, and 0.75–0.90: Good reliability (Liljequist et al., 2019).

## Results

### Effects of SNR on spatial release from masking and pupil dilation

**Figure 1:**
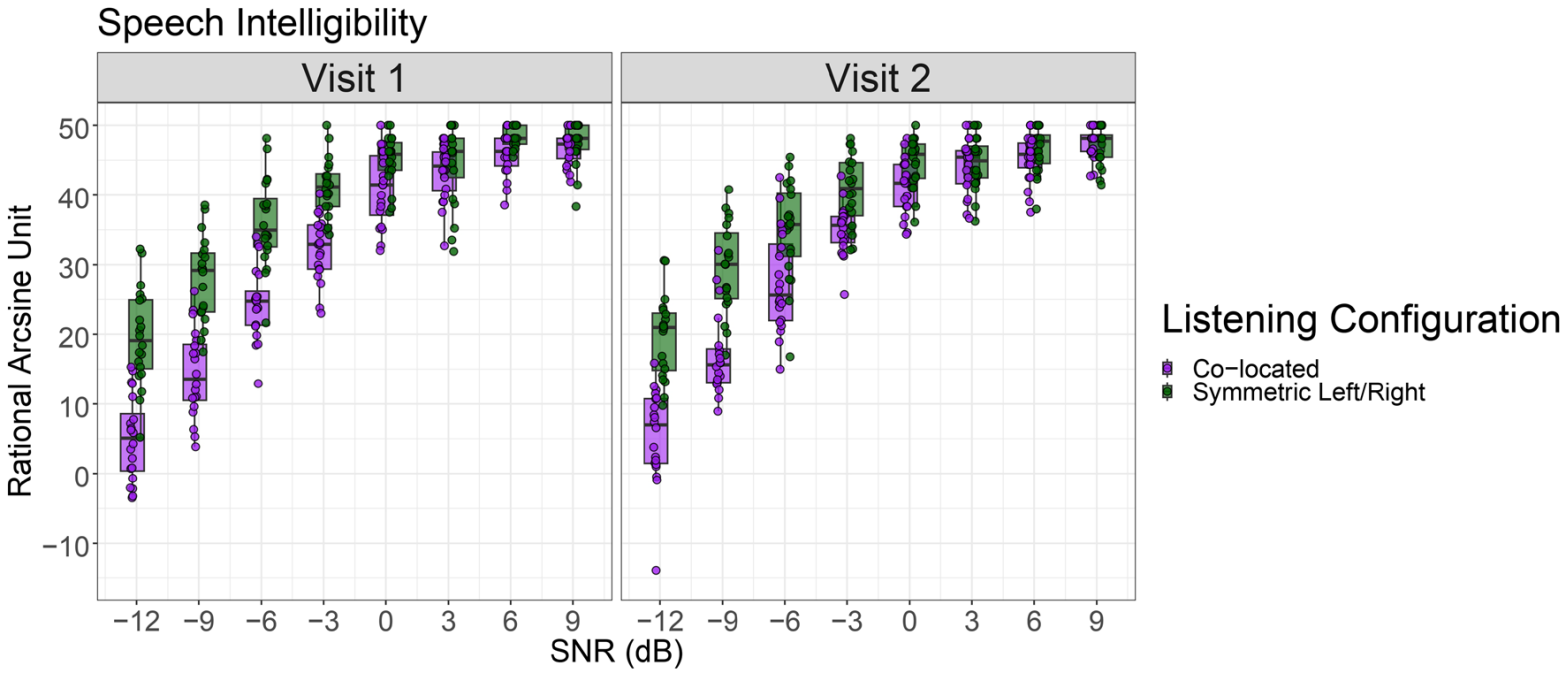
Speech intelligibility scores (in rational arcsine units) as a function of signal-to-noise ratio (SNR, dB) across two visits. Results are shown separately for co-located and symmetric left/right listening configurations. The boxes represent the range of the first and third quartiles, while whiskers extend to 1.5 times the inter quartile range.

**Figure 2:**
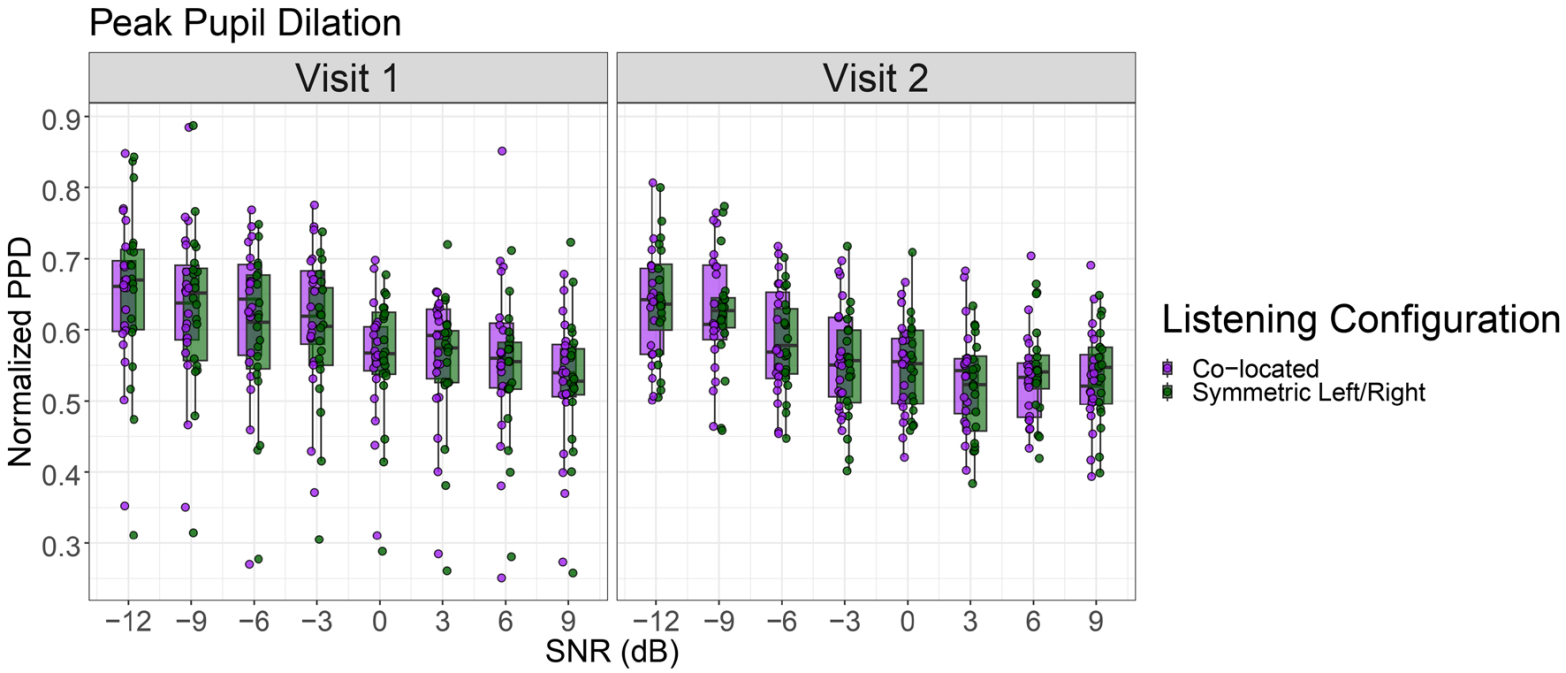
Range-normalized and baseline-corrected change in peak pupil dilation (PPD) as a function of signal-to-noise ratio (SNR, dB) across two visits and listening configurations. Data are shown for co-located and symmetric left/right listening conditions. The boxes represent the range of the first and third quartiles, while whiskers extend to 1.5 times the inter quartile range.

Consistent with our first hypothesis, participants demonstrated robust speech intelligibility (RAU) and PPD in the symmetric left/right configuration compared to the co-located configuration across SNR levels. This pattern is evident in Figure 1, which shows the group-level relationship between SNR and PPD, and in Figure 2, which further demonstrates the SNR-dependent benefit, in line with our predictions about spatial hearing advantages in noisy environments. For the speech intelligibility model, we found there were significant main effects of SNR, *F*(1, 613) = 2407.30, *p* < 0.001, and Listening Configuration, *F*(1, 613) = 124.12, *p* < 0.001, on speech intelligibility scores. The SNR and Listening Configuration interaction was also significant, *F*(1, 613) = 120.91, *p* < 0.001. However, neither test session (i.e., Visit 1 or Visit 2) nor its interactions reached significance (all *p*s > 0.05), suggesting that overall speech intelligibility performance did not differ between visits. The inclusion of a random intercept revealed the presence of small but meaningful baseline differences in RAU across individuals; scaled residuals ranged from −4.22 to 2.52, The variance of the random intercept was 5.66 (*SD* = 2.38), and residual variance was 33.45 (*SD* = 5.78) across 640 observations from 20 participants.

Model coefficients revealed that higher SNRs were associated with improved RAU scores (*β* = 2.04, *p* < 0.001). The symmetric left/right (spatially separated) Listening Configuration yielded significantly better performance than the co-located condition (*β* = 5.95, *p* < 0.001). There was a significant interaction between SNR and Listening Configuration (*β* = −0.74, *p* < 0.001), reflecting a reduced spatial benefit at higher SNRs. There were no significant effects of test session (Visit Number) and no higher-order interactions involving test session (Table 2).

**Table 2:** Fixed effects estimates from the linear mixed-effects model predicting speech intelligibility scores (RAU).

Predictor	Estimate	SE	DF	*t*	*p*
Intercept	34.72	0.71	42.26	48.99	<0.001***
SNR	2.04	0.07	613	30.7	<0.001***
Listening configuration (symmetric left/right)	5.95	0.66	613	8.99	<0.001***
Visit 2	1.1	0.66	613	1.66	0.099
SNR × Listening configuration	−0.74	0.09	613	−7.92	<0.001***
SNR × Visit 2	−0.09	0.09	613	−0.95	0.344
Listening configuration × Visit 2	−1.48	0.94	613	−1.58	0.114
SNR × Listening configuration × Visit 2	0.03	0.13	613	0.2	0.844

Significance codes: ***p < 0.001, **p < 0.01, *p < 0.05.

**Figure 3:**
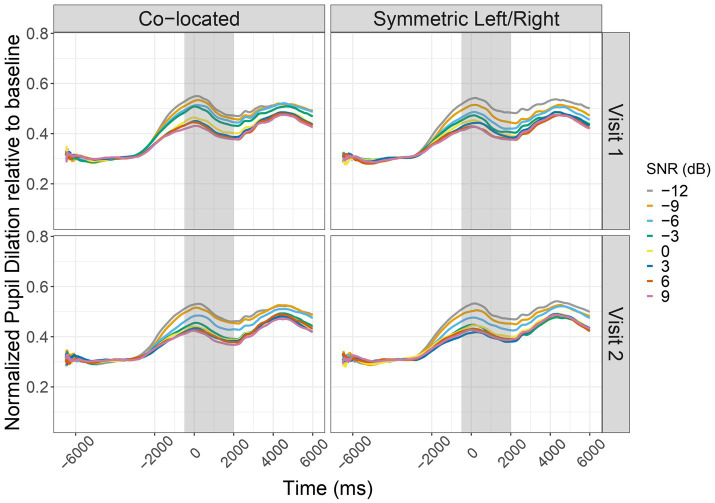
Range normalized and baseline-corrected pupil dilation averaged across participants plotted as a function of time relative to the stimulus’ offset. Each pupil track represents a different Signal-to-Noise Ratio (SNR) across Co-located and Symmetric Left/Right spatial conditions, shown separately for Visit 1 (top row) and Visit 2 (bottom row). Colored lines represent SNR levels from -12 dB to +9 dB. The shaded region represents the response window where peak pupil dilation (PPD) was extracted.

Regarding listening effort, we found that there were significant main effects of SNR, *F*(1, 613) = 266.54, *p* < 0.001, and Visit Number, *F*(1, 613) = 12.47, *p* < 0.001, on PPD. In comparison to speech intelligibility, there was no main effect of Listening Configuration, and all interaction terms were also non-significant (all *p* > 0.05). Listening effort, as measured by PPD, decreased in easier listening conditions indicating that a decrease in task difficulty was associated with lower PPD (*β* = −0.0058, *p* < 0.001), consistent with prior research (Zekveld et al., 2018; Zekveld et al., 2013). There was no significant advantage for the symmetric left/right configuration (*β* = −0.0109, *p* = 0.111), and no evidence of interaction effects among predictors. Between-subject variance in baseline PPD was small compared to within-subject residual variance. A random intercept for participant captured baseline differences in PPD across individuals, with an estimated variance of 0.0048 (*SD* = 0.069). The within-subjects residual variance was 0.0035 (*SD* = 0.059). The sample comprised 640 observations from 20 participants (Figure 3; Table 3).

**Table 3:** Fixed effects estimates for the linear mixed-effects model predicting peak pupil dilation.

Predictor	Estimate	SE	DF	*t*	*p*
Intercept	0.582	0.016	21.82	35.85	<0.001***
SNR	−0.0058	0.0007	613	−8.52	<0.001***
Listening configuration (symmetric left/right)	−0.0109	0.0068	613	−1.60	0.111
Visit 2	−0.0222	0.0068	613	−3.27	0.001**
SNR × Listening configuration	−0.0001	0.0010	613	−0.11	0.911
SNR × Visit 2	0.0005	0.0010	613	0.47	0.637
Listening configuration × Visit 2	0.0105	0.0096	613	1.09	0.278
SNR × Listening configuration × Visit 2	0.0003	0.0014	613	0.21	0.836

Significance codes: ***p < 0.001, **p < 0.01, *p < 0.05.

Listening effort results demonstrate that PPD decreased with increasing SNR, and were reduced on the second visit but showed no effect of Listening Configuration. The configuration effect was also influenced by test session, but no other interactions reached statistical significance. However, although the observed coefficients were statistically significant, the small magnitudes of them reflects a minimal effect unlikely to be of practical importance. Thus, we caution against overinterpreting statistical findings in absence of substantial effect sizes.

### Effects of test session on speech intelligibility and PPD

Test–retest reliability across session (Visit 1 vs. Visit 2) was assessed using single-score intraclass correlation coefficients (ICCs), computed under a two-way mixed-effects ICCs calculated using data aggregated by participant, and SNR, reflecting average performance across trials. Overall, the ICC for RAU scores in the symmetric left/right configuration across Visit 1 and Visit 2 sessions was high, ICC (2,1) = 0.86, 95% CI [0.81, 0.89], *F*(159, 159) = 12.90, *p* < 0.001. For the co-located configuration, the ICCs indicated even higher reliability, ICC (2,1) = 0.93, 95% CI [0.91, 0.95], *F*(159, 159) = 29.20, *p* < 0.001. ICCs for in the co-located configuration ranged from poor to moderate, with ICCs ranging from −0.03 to 0.58. ICCs for the symmetric left/right configuration ranged from −0.13 to 0.62 (see Table 4). For example, in the co-located configuration at −6 dB SNR, ICC = 0.58, whereas at 0 dB SNR, ICC = 0.05. In the symmetric left/right configuration at 9 dB SNR, reliability was highest (ICC = 0.62).

**Table 4:** RAU intraclass correlation coefficients across all listening configurations and SNRs.

Listening configuration	SNR	ICC
Co-located	−12	0.42
Co-located	−9	0.43
Co-located	−6	0.58
Co-located	−3	0.32
Co-located	0	0.05
Co-located	3	−0.03
Co-located	6	0.42
Symmetric	−12	0.33
Symmetric	−9	0.33
Symmetric	−6	0.37
Symmetric	−3	0.47
Symmetric	0	0.25
Symmetric	3	0.22
Symmetric	6	−0.13
Symmetric	9	0.62

Reliability for listening effort, represented by PPD, were computed using data aggregated at the participant and SNR level, comparing Visit 1 and Visit 2. For the co-located condition, reliability was moderate, ICC (2,1) = 0.53, 95% CI [0.41, 0.63], *F*(159, 159) = 3.22, *p* < 0.001. For the symmetric left/right configuration, reliability was also moderate, ICC (2,1) = 0.55, 95% CI [0.44, 0.65], *F*(159, 159) = 3.49, *p* < 0.001. When analyzed by Listening Configuration and SNR, PPD test–retest reliability values ranged from poor to moderate (see Table 5). In the co-located configuration, ICCs ranged from 0.11 (6 dB SNR) to 0.51 (−9 dB SNR). In the symmetric left/right configuration, ICCs ranged from 0.20 (3 dB SNR) to 0.37 (−3 dB SNR). The quiet condition yielded an ICC of 0.40. These findings indicated that reliability measures, while improved when aggregated, showed poorer reliability than the speech intelligibility scores.

**Table 5:** PPD intraclass correlation coefficients across all listening configurations and SNRs.

Listening configuration	SNR	ICC
Co-located	−12	0.487
Co-located	−9	0.506
Co-located	−6	0.311
Co-located	−3	0.306
Co-located	0	0.317
Co-located	3	0.273
Co-located	6	0.111
Symmetric left/right	−12	0.209
Symmetric	−9	0.337
Symmetric left/right	−6	0.367
Symmetric left/right	−3	0.373
Symmetric left/right	0	0.274
Symmetric left/right	3	0.195
Symmetric left/right	6	0.204
Symmetric left/right	9	0.324

## Discussion

The primary objective of this study was two-fold: to evaluate the impact of target-masker spatial separation on listening effort, as a function of SNR, through measures of speech intelligibility and pupil dilation, and to assess the reliability of speech intelligibility and pupillometric measures over two testing sessions. We focused on individuals with typical hearing to explore how spatial separation of sound sources affects speech intelligibility and PPD, the latter being a representation of listening effort.

### Impact of spatial separation on listening effort

Early research by Broadbent (1954) demonstrated that separating sound sources spatially can enhance the accuracy of responses, as the auditory system leverages spatial cues provided by binaural hearing. Findings from Broadbent (1954) suggested a possible involvement of distinct cognitive processes when individuals are asked to discriminate between different sound source locations. Similarly, our study provides further insights into how factors such as SNR and spatial location shape individual differences that could enhance a listener’s ability to focus attention and improve the detection, segregation, and recognition of sounds. A key finding of the present study was that spatial separation led to higher speech intelligibility, but did not produce a corresponding reduction in listening effort, and this effect was modest. This suggests that while spatial separation systematically enhances speech intelligibility, its effects on physiological measures such as pupil dilation are complex and may require larger sample sizes to detect reliably within an SRM paradigm.

Previous authors have attempted to implicate cognitive processes such as attention in a wide range of spatial hearing paradigms. For instance, prior work demonstrates that spatial unmasking depends on both energetic masking and informational masking, the latter relying on attentional mechanisms, such as the ability to focus on spatially separate sounds (Shinn-Cunningham et al., 2005). A critical finding from Shinn-Cunningham et al. (2005) was that spatial unmasking at lower target-to-masker ratios (TMRs) primarily relies on energetic masking, while higher TMRs engage attentional processes. Papesh et al. (2017) showed that auditory evoked potentials measuring cortical responses to interaural phase differences, were highly predictive of participants’ SRM performance. These cortical measures of binaural sensitivity were found to be better predictors of speech understanding in noisy environments than age or hearing loss alone. Furthermore, informational masking has been found to have a more substantial impact than energetic masking in situations where the target and masker voices are similar, highlighting the importance of cognitive load and attention in the ability to segregate competing speech signals for understanding in multi-talker environments for those with typical hearing (Brungart et al., 2001; Koelewijn et al., 2012), or with hearing impairment (Stenbäck et al., 2022). This is especially true under spectrally degraded conditions, where prior research has revealed the impact of informational masking even when TH listeners are exposed to CI-simulated speech, emphasizing the necessity of preserving these cues in auditory prostheses such as cochlear implants in SRM paradigms (Garadat et al., 2009).

Together, these studies suggest that the engagement of higher-order attentional processes may depend on the SNR. This further highlights the importance of our current study’s goals to investigate both the behavioral and physiological ramifications of SRM. Yet, it should be acknowledged that manipulation of experimental parameters like SNR and masker configuration, are intended to modulate cognitive load and task difficulty, which in turn engage attentional and effortful listening mechanisms, rather than being cognitive processes themselves. In the present study, since only a two-talker masker was employed, we cannot make substantial claims that participants were relying on attention mechanisms from informational masking, however, future studies should consider investigating cognitive differences associated with energetic or informational masking in an SRM paradigm.

A key takeaway from previous studies is that, while behavioral paradigms are essential for understanding binaural mechanisms, an individual’s ability to detect sounds accurately and consistently is strongly influenced by the contextual information present in the test materials. Findings from our reliability measures showed that neither speech intelligibility nor pupil dilation measures revealed extremely high levels of agreement across test sessions, suggesting that the interpretation of data from SRM paradigms that incorporate pupillometric measures, while meaningful, may only be relevant within a single test session. For future studies, researchers should consider using a broader range and types of test paradigms that manipulate additional contextual cues, such as informational vs. energetic masking or introducing spectral degradation of binaural cues. While incorporating these factors may increase the complexity of the experimental design, doing so can enhance ecological validity and provide a more accurate reflection of one’s ability to focus and attend to target sounds in real-world, multi-talker environments.

### Reliability of measures across sessions

In the present study we also used reliability measures to assess the consistency of our data across two separate test sessions. Here, we employed a hypothesis-driven approach to evaluate reliability, focusing on how well a measurement remains consistent over time (Bland and Altman, 1986; Mokkink et al., 2023). We found that speech intelligibility, measured using RAU scores, while showing variations across different SNRs and Listening Configurations, showed moderate-to-high reliability between the two visits. This suggests that speech intelligibility, while possibly influenced by task difficulty or participant attention, is still a reliable measure of SRM. In contrast, the PPD measures showed less robust agreement across the visits, implying that reliability of listening effort appears to be less stable, compared to speech intelligibility measures, across two different time points, even when the auditory environments are replicated. We also observed a greater spread in PPD during Visit 1 compared to Visit 2 (see Figure 2) which likely reflects a combination of task novelty, individual differences in initial engagement, or greater between-subject variability at the first session, further emphasizing the need for larger sample sizes and more multi-session testing. Additionally, research indicates that pupil dilation tends to decrease as participants become more familiar with a task or experience mental fatigue (McGarrigle et al., 2014; Papesh et al., 2012). Recent studies assessing speech-in-noise have demonstrated that baseline-corrected and normalized pupil dilation offers the highest reliability (Neagu et al., 2023). This study found that SNR and the number of visits has only a modest impact on data reliability. Although we followed Neagu’s normalization approach, we recommend interpreting the reliability of our results with caution given the inherent variability in participant factors.

### Implication for future research and clinical applications

The moderate-to-low reliability in PPD and the moderate-to-high reliability in RAU scores across two test sessions highlight the complexities involved in auditory processing, particularly in noise. For individuals with hearing loss, particularly cochlear implant users, understanding the link between cognitive effort (as indexed by pupil dilation) and spatial hearing performance is critical. At present, our findings indicate that improvements in speech intelligibility for symmetric, spatially separated configurations do not correspond with decreases in pupil dilation across test sessions. This suggests that validating PPD as a reliable cognitive load measure in binaural paradigms like SRM will necessitate larger-scale studies to assess its potential utility as a tool for auditory rehabilitation in listeners with bilateral devices. For instance, individual factors such as aging can create substantial barriers impacting binaural processing, resulting in reduced SRM performance in individuals with hearing loss (Papesh et al., 2017). Overall, reducing cognitive load should be prioritized because individuals with hearing impairments often experience increased mental fatigue when trying to comprehend speech in challenging listening environments (Alhanbali et al., 2017; McGarrigle et al., 2014). It should be noted that SRM paradigms are intended to measure benefits from spatial separation of sounds sources (see Suveg et al., 2025); in the present study we have aimed to measure any reductions in cognitive load associated with spatial separation, rather than suggest that implementing binaural hearing will reduce cognitive load overall. Furthermore, SRM paradigms, like the one used in the present study, is not suited for clinical use due to practical considerations, however, these findings contribute to a growing body of research revealing the mechanisms underlying spatial hearing and listening effort combined with reliability measures. Future work should leverage these findings to inform the development of time-efficient, clinically feasible assessments, provided that sufficient effect sizes and reliability are established.

### Limitations and future directions

Our study provided valuable insights; however, several aspects could be enhanced in future research. A key objective of this study was to complete an investigation on the reliability of data for binaural test paradigms such as SRM, when paired with physiological measures, such as pupil dilation. One limitation was that ceiling effects for RAU scores at higher SNRs may have reduced sensitivity to differences in speech intelligibility measures, constraining interpretability in easier conditions. We also determined that, while these measures may have value for determining binaural outcomes, the interpretations of speech intelligibility paired with listening effort measures must be handled with caution. For instance, the current study found moderate-to-low levels of reliability for PPD across sessions, regardless of Listening Configuration. Thus, it is important for future SRM studies to demonstrate higher reliability of pupillometric listening effort measures. Prior studies such as, Alhanbali et al. (2017) and Giuliani et al. (2021) reported good to excellent ICCs for PPD between repeated test sessions. It is very possible that the relatively modest ICCs observed in our study may partially reflect the extended session length and participant fatigue, as our test protocol required approximately 6 h per session. Fatigue is well-documented to attenuate pupillary responses over time (e.g., Zekveld et al., 2018) and can reduce between-session consistency (Neagu et al., 2023; Winn et al., 2018). Thus, we acknowledge that pupillometric measures of listening effort can, under more constrained or optimized protocols, achieve high statistical reliability We also note that negative ICC values were determined when comparing speech intelligibility (RAU) between sessions. Negative ICCs generally indicate poor agreement, often reflecting that within-subject variability exceeds between-subject variability or that there are baseline differences across participants. In our data, these negative values were limited to +3 and −6 dB SNR, with ICCs of −0.03 and −0.13, respectively. Altogether, these findings further emphasize the need to incorporate variables such as session duration, participant state, and protocol design when interpreting test–retest reliability in SRM paradigms combined with pupillometric measures.

Future research should aim to include a larger and more diverse sample and collect data prior to the onset of participant fatigue to strengthen the validity and generalizability of the findings. Additionally, incorporating more realistic and varied auditory environments could help clarify the benefits of spatial separation in everyday listening situations. Finally, further studies should explore the longitudinal reliability of these measures to better understand their implications for long-term auditory rehabilitation. In conclusion, while our study provides valuable insights into the reliability of speech intelligibility and pupillometric measures in a controlled environment, these findings do not support any direct clinical application or intervention. Our use of young, typical hearing participants and non-clinical stimuli means results are only applicable in research contexts. The present study also highlights the need for further research to explore these dynamics in more complex and variable conditions.

## Data Availability

The raw data supporting the conclusions of this article will be made available by the authors, without undue reservation.

## Author’s note

Portions of this work were presented at the Association for Research in Otolaryngology Mid-Winter Meeting, Baltimore, MD, July 2019.
